# Supplementary material for: Prospective associations between coffee consumption and psychological well-being
Source: PLoS One. 2022 Jun 9;17(6):e0267500. doi: 10.1371/journal.pone.0267500 (PMC9182697; doi:10.1371/journal.pone.0267500)
Supplement: S1 File — (DOCX) [file pone.0267500.s001.docx]

**Online Supplement**

**Supplemental Figure S1.** *Timeline of Coffee and Psychological Well-being Assessments Within Each Analytic Sample*

**
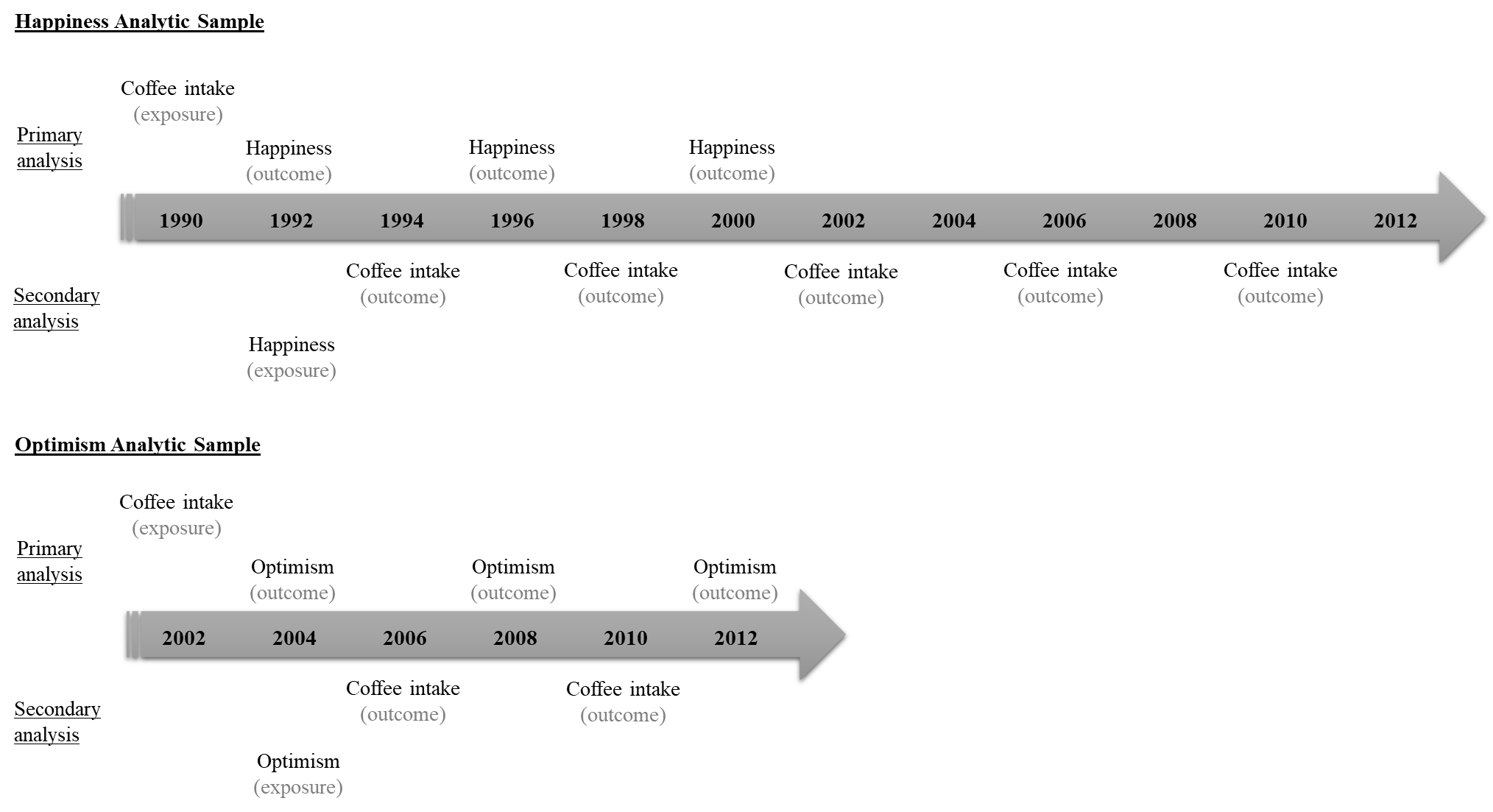
**

**Supplemental Table S1.** *Comparison of Study Covariates Among Women Who Were Included Versus Excluded from the Study Based on Their Completion of the Coffee Consumption Measure at Each Analytic Baseline.^a,b^*

|  | **Included**  **(n=79,268)** | **Excluded**  **(n=42,433)** |
| --- | --- | --- |
| **Analytic Baseline for Happiness Analyses (1990)** | Percent or  Mean (SD) | Percent or  Mean (SD) |
| Age, *Years* | 56.50 (7.16) | 56.79 (7.30) |
| White, % | 97.78 | 94.91 |
| Graduate degree, % | 9.91 | 8.28 |
| Married/in a relationship, % | 81.76 | 78.74 |
| ≥1 chronic health condition, % | 18.75 | 30.39 |
| Depressed, % | 7.65 | 3.84 |
| Socially integrated, % | 25.70 | 23.84 |
| Lifestyle Index, *Continuous Score* | 2.31 (1.20) | 2.46 (1.20) |
| Happiness, *Continuous Score* | 4.52 (0.95) | 4.44 (0.99) |
|  | **Included**  **(n=79,242)** | **Excluded**  **(n=42,459)** |
| **Analytic Baseline for Optimism Analyses (2002)** | Percent or  Mean (SD) | Percent or  Mean (SD) |
| Age, *Years* | 67.91 (7.05) | 69.86 (7.36) |
| White, % | 97.58 | 95.29 |
| Graduate degree, % | 10.02 | 8.02 |
| Married/in a relationship, % | 73.32 | 65.46 |
| ≥1 chronic health condition, % | 34.32 | 66.83 |
| Depressed, % | 13.21 | 4.43 |
| Socially integrated, % | 26.85 | 24.13 |
| Lifestyle Index, *Continuous Score* | 2.31 (1.19) | 2.24 (1.14) |
| Optimism, *Continuous Score* | 24.67 (4.56) | 24.04 (4.86) |
| ^a^ Characteristics were measured at each analytic baseline (i.e., 1990 or 2002), unless otherwise specified in the Methods section.  ^b^ SD=standard deviation. | | |
|  |  |  |

**Supplemental Table S2.** *Comparison of Women Included in the Analytic Samples by Their Availability of Psychological Well-being Data at Follow-up Assessments.^a^*

|  | **No Missing  Happiness Data**  **(n=40,320)** | **Some Missing**  **Happiness Data**  **(n=4,129)** |
| --- | --- | --- |
| **Happiness Analytic Sample (1990-2000)** | Percent or  Mean (SD) | Percent or  Mean (SD) |
| Age, *Years* | 55.85 (7.00) | 56.92 (7.56) |
| White, % | 98.34 | 97.36 |
| Graduate degree, % | 10.63 | 9.18 |
| Married/in a relationship, % | 83.81 | 78.83 |
| Depressed, % | 7.35 | 9.06 |
| Socially integrated, % | 26.63 | 23.35 |
| Lifestyle Index, *Continuous Score* | 2.40 (1.20) | 2.19 (1.20) |
| Happiness, *Continuous Score* | 4.56 (0.92) | 4.48 (0.97) |
|  | **No Missing**  **Optimism Data**  **(n=31,044)** | **Some Missing**  **Optimism Data**  **(n=5,685)** |
| **Optimism Analytic Sample (2002-2012)** | Percent or  Mean (SD) | Percent or  Mean (SD) |
| Age, *Years* | 65.90 (6.53) | 70.35 (6.94) |
| White, % | 98.20 | 97.91 |
| Graduate degree, % | 11.23 | 8.16 |
| Married/in a relationship, % | 78.65 | 69.08 |
| Depressed, % | 10.46 | 11.96 |
| Socially integrated, % | 29.47 | 23.76 |
| Lifestyle Index, *Continuous Score* | 2.46 (1.20) | 2.28 (1.18) |
| Optimism, *Continuous Score* | 25.19 (4.42) | 24.31 (4.50) |

^a^ SD=standard deviation.

| **Supplemental Table S3.** *Generalized Estimating Equations with a Poisson Distribution Evaluating the Association of Baseline Alternative Beverages and Caffeine-Related Intake with Likelihood of Reporting Sustained High Levels of Happiness (Ns=43,505 to 44,449).^a,b,c^* | | | | |  |
| --- | --- | --- | --- | --- | --- |
|  | **Sustained High Happiness Between 1992 and 2000** | | | | |
|  | Model 1 | Model 2 | Model 3 | Model 4 | |
|  | RR (95% CI) | RR (95% CI) | RR (95% CI) | RR (95% CI) | |
|  |  | | | | |
| **Decaffeinated Coffee Intake in 1990** | | |  |  | |
| Less than 1 cup/day | Reference | Reference | Reference | Reference | |
| 1-3 cups/day | 1.01 (0.99-1.02) | 1.00 (0.98-1.01) | 0.99 (0.98-1.00) | 0.99 (0.98-1.01) | |
| 4 or more cups/day | 1.00 (0.97-1.04) | 1.00 (0.96-1.03) | 0.99 (0.96-1.02) | 1.00 (0.96-1.03) | |
| *Linear p-trend* |  |  |  | 0.28 | |
| **Caffeinated Tea Intake in 1990** | | |  |  | |
| Less than 1 cup/day | Reference | Reference | Reference | Reference | |
| 1-3 cups/day | 1.00 (0.99-1.02) | 1.00 (0.99-1.02) | 1.00 (0.98-1.01) | 0.99 (0.98-1.01) | |
| 4 or more cups/day | 0.98 (0.94-1.01) | 0.98 (0.95-1.02) | 0.99 (0.95-1.02) | 0.99 (0.95-1.02) | |
| *Linear p-trend* |  |  |  | 0.28 | |
| **Overall Caffeine Intake in 1990** |  |  |  |  | |
| Less than 100 mg/day | Reference | Reference | Reference | Reference | |
| 100-549 mg/day | 0.98 (0.96-0.99)^***^ | 0.98 (0.97-0.99)^**^ | 0.99 (0.98-1.00) | 0.99 (0.97-1.00)^*^ | |
| 550 or more mg/day | 0.94 (0.92-0.96)^****^ | 0.96 (0.94-0.98)^****^ | 0.98 (0.96-1.00)^*^ | 0.97 (0.95-0.99)^***^ | |
| *Linear p-trend* |  |  |  | 0.001 | |
| ^a^ Sample sizes for these analyses vary because there were missing data on the decaffeinated coffee and caffeinated tea exposures. | | | | | |
| ^b^ Sociodemographic covariates included in Model 1 include age, race, education, and marital status. Model 2 additionally includes information on health behaviors compiled into a single lifestyle score that includes BMI, diet quality [AHEI], alcohol consumption, physical activity and smoking. Model 3 further adjusts for social integration. Fully adjusted Model 4 includes covariates from all prior models, plus depression defined using physician diagnoses and antidepressant use.  ^c^ CI=confidence interval; RR= relative risk.  *****p≤*0.0001; ****p≤*0.001; ***p≤*0.01; **p≤*0.05 | | | | | |

| **Supplemental Table S4.** *Generalized Estimating Equations with a Poisson Distribution Evaluating the Association of Baseline Alternative Beverages and Caffeine-Related Intake with Likelihood of Reporting Sustained High Levels of Optimism (Ns=36,213 to 36,729)^a,b,c^* | | | | |  |
| --- | --- | --- | --- | --- | --- |
|  | **Sustained High Optimism Between 2004 and 2012** | | | | |
|  | Model 1 | Model 2 | Model 3 | Model 4 | |
|  | RR (95% CI) | RR (95% CI) | RR (95% CI) | RR (95% CI) | |
|  |  | | | | |
| **Decaffeinated Coffee Intake in 2002** | | |  |  | |
| Less than 1 cup/day | Reference | Reference | Reference | Reference | |
| 1-3 cups/day | 1.00 (0.96-1.03) | 0.98 (0.94-1.02) | 0.96 (0.93-1.00)^*^ | 0.96 (0.93-1.00)^*^ | |
| 4 or more cups/day | 1.04 (0.93-1.17) | 1.04 (0.93-1.17) | 1.03 (0.92-1.15) | 1.02 (0.91-1.14) | |
| *Linear p-trend* |  |  |  | 0.09 | |
| **Caffeinated Tea Intake in 2002** | | |  |  | |
| Less than 1 cup/day | Reference | Reference | Reference | Reference | |
| 1-3 cups/day | 0.97 (0.93-1.01) | 0.96 (0.92-1.00) | 0.97 (0.93-1.01) | 0.96 (0.92-1.01) | |
| 4 or more cups/day | 0.99 (0.88-1.13) | 0.99 (0.88-1.13) | 1.00 (0.89-1.14) | 1.01 (0.89-1.14) | |
| *Linear p-trend* |  |  |  | 0.16 | |
| **Overall Caffeine Intake in 2002** |  |  |  |  | |
| Less than 100 mg/day | Reference | Reference | Reference | Reference | |
| 100-549 mg/day | 1.01 (0.98-1.04) | 1.01 (0.98-1.05) | 1.03 (1.00-1.06)^**^ | 1.03 (1.00-1.06)^*^ | |
| 550 or more mg/day | 0.96 (0.83-1.11) | 1.00 (0.87-1.16) | 1.03 (0.90-1.19) | 1.02 (0.89-1.17) | |
| *Linear p-trend* |  |  |  | 0.09 | |
| ^a^ Sample sizes for these analyses vary because there were missing data on the decaffeinated coffee and caffeinated tea exposures. | | | | | |
| ^b^ Sociodemographic covariates included in Model 1 include age, race, education, and marital status. Model 2 additionally includes information on health behaviors compiled into a single lifestyle score that includes BMI, diet quality [AHEI], alcohol consumption, physical activity and smoking. Model 3 further adjusts for social integration. Fully adjusted Model 4 includes covariates from all prior models, plus depression defined using physician diagnoses and antidepressant use.  ^c^ CI=confidence interval; RR= relative risk.  *****p≤*0.0001; ****p≤*0.001; ***p≤*0.01; **p≤*0.05 | | | | | |

| **Supplemental Table S5.** *Generalized Estimating Equations with a Poisson Distribution Evaluating the Bidirectional Associations Between Coffee Intake and Happiness/Optimism, While Adjusting for Baseline Level of the Outcome (Ns=31,441 to 44,449)^a,b,c^* | | | | |  |
| --- | --- | --- | --- | --- | --- |
|  | Model 1 | Model 2 | Model 3 | Model 4 | |
|  | RR (95% CI) | RR (95% CI) | RR (95% CI) | RR (95% CI) | |
| **Coffee Intake in 1990 with Sustained High Happiness Between 1996 and 2000** | | |  |  | |
| Less than 1 cup/day | Reference | Reference | Reference | Reference | |
| 1-3 cups/day | 1.02 (1.00-1.03) | 1.01 (1.00-1.03) | 1.02 (1.00-1.04)^**^ | 1.02 (1.00-1.03)^*^ | |
| 4 or more cups/day | 0.97 (0.94-1.00)^*^ | 0.98 (0.96-1.01) | 0.99 (0.96-1.02) | 0.98 (0.96-1.01) | |
| **Coffee Intake in 2002 with Sustained High Optimism Between 2008 and 2012** | | |  |  | |
| Less than 1 cup/day | Reference | Reference | Reference | Reference | |
| 1-3 cups/day | 1.02 (0.98-1.06) | 1.02 (0.97-1.06) | 1.02 (0.98-1.07) | 1.02 (0.98-1.06) | |
| 4 or more cups/day | 1.00 (0.91-1.10) | 1.02 (0.93-1.12) | 1.03 (0.93-1.13) | 1.02 (0.93-1.12) | |
| **Happiness in 1992 with Sustained Moderate Coffee Intake Between 1998 and 2010** | | |  |  | |
| Low | Reference | Reference | Reference | Reference | |
| Moderate | 1.00 (0.97-1.03) | 0.99 (0.96-1.02) | 0.99 (0.96-1.02) | 0.99 (0.96-1.02) | |
| High | 1.04 (1.01-1.07)^**^ | 1.03 (1.00-1.05)^*^ | 1.03 (1.01-1.06)^**^ | 1.03 (1.00-1.05)^*^ | |
| **Optimism in 2004 with Sustained Moderate Coffee Intake in 2010 Only ^d^** | | | | | |
| Low | Reference | Reference | Reference | Reference | |
| Moderate | 1.02 (0.98-1.06) | 1.02 (0.98-1.06) | 1.02 (0.98-1.06) | 1.02 (0.98-1.06) | |
| High | 1.03 (0.99-1.07) | 1.02 (0.99-1.06) | 1.03 (0.99-1.07) | 1.03 (0.99-1.07) | |
| ^a^ Sample sizes for these analyses vary because there were missing data on the decaffeinated coffee and caffeinated tea exposures. | | | | | |
| ^b^ Sociodemographic covariates included in Model 1 include age, race, education, and marital status. Model 2 additionally includes information on health behaviors compiled into a single lifestyle score that includes BMI, diet quality [AHEI], alcohol consumption, physical activity and smoking. Model 3 further adjusts for social integration. Fully adjusted Model 4 includes covariates from all prior models, plus depression defined using physician diagnoses and antidepressant use.  ^c^ CI=confidence interval; RR= relative risk.  ^d^ Tests of associations between optimism and subsequent coffee intake only used coffee intake data from one follow-up point, therefore models were evaluated using logistic regression.  *****p≤*0.0001; ****p≤*0.001; ***p≤*0.01; **p≤*0.05 | | | | | |

**Supplemental Text S1**

***Creation of the Lifestyle Score***

Consistent with a lifestyle index used in previous studies (Loef & Walach, 2012; Trudel-Fitzgerald, James, et al., 2019; Trudel-Fitzgerald, Tworoger, Poole, Williams, & Kubzansky, 2016) as well as with available cancer and cardiovascular guidelines (Kushi et al., 2012; Mosca et al., 2011), the lifestyle score included five behavior-related factors: physical activity, diet, body mass index (BMI), alcohol and tobacco consumption. Individual factors were obtained via self-report at or within two years of each analytic baseline (happiness analytic sample: 1990; optimism analytic sample: 2002). Based on prior research (Grimmett, Bridgewater, Steptoe, & Wardle, 2011; Loef & Walach, 2012; Schlesinger et al., 2014; Trudel-Fitzgerald, James, et al., 2019), behavior-related factors were first dichotomized according to whether individuals were compliant with recommended guidelines or not (1/0) and then summed to create a lifestyle score, ranging from 0 “least healthy” to 5 “most healthy.”

Physical activity was assessed with a validated self-administered questionnaire (Chasan-Taber et al., 1996). It measures the average weekly time spent at various activities, from inactive to vigorous ones, over the past year. The questionnaire has shown high validity when compared against 1-week diaries (e.g., correlation with vigorous activities, *r* values from 0.54 to 0.58) and resting pulse rate as an objective fitness metric independent of recall (e.g., correlation with vigorous activities, *r* values from -0.41 to -0.45). Reproducibility over a one-year period was found to be adequate as well (e.g., for vigorous activities, intra-class correlation [ICC] values from 0.32 to 0.79). In the current study, a score of 1 was assigned when women reported ≥150 minutes per week of moderate-to-vigorous activity (e.g., brisk walking, running, bicycling). BMI was derived using women’s self-reported initial height and updated weight. Previous work with the cohort has shown self-reported weight is highly correlated with weight measured by study staff (*r*=0.96) (Rimm et al., 1990). Healthy weight (score of 1) was defined as BMI≤25 kg/m^2^. Dietary information was obtained from the 131-item Food Frequency Questionnaire, which has high reproducibility and validity when compared with 1-week diet records and biochemical markers (Giovannucci et al., 1991; Rimm et al., 1992). The summary score used here encompasses the following components of the Alternative Healthy Eating Index (McCullough et al., 2002): higher intake of vegetables, fruit, whole grains, nuts and legumes, long-chain (n-3) fatty acids, polyunsaturated fats; lower intake of sugar-sweetened beverages and fruit juice, red/processed meat, saturated fats, sodium. The score for each component ranged from 0 to 10 (optimal dietary behavior). Within this cohort, researchers have used the highest 40% of the diet score distribution as a cutoff to indicate healthy diet (Chiuve et al., 2008; Stampfer, Hu, Manson, Rimm, & Willett, 2000), and found it is related to a lower risk of several diseases, including stroke, diabetes and cancer (Chiuve et al., 2012). Accordingly, a healthy diet (score of 1) was defined as a score in the top 40% of the current cohort distribution. Healthy alcohol consumption (score of 1) was defined as drinking on average 1 drink/day (Kushi et al., 2012; Mosca et al., 2011). Lastly, women received a score of 1 if they reported currently being a non-smoker.

**Supplemental Text S2**

***Social Integration Measure***

Social networks were assessed at or close to each analytic baseline (happiness analytic

sample: 1992; optimism analytic sample: 2004) with the Berkman-Syme Social Network Index (Berkman & Syme, 1979), a multidimensional measure that has been widely used in prior research (Holt-Lunstad, Robles, & Sbarra, 2017). This index assesses four distinct dimensions of social networks: marital status (married/partnered, separated/divorced, widowed); number of close relatives and close friends, separately (0, 1–2, 3–5, 6–9, ≥10); frequency of religious activities (>1/week, 1/week, 1/month–1/year, never); and frequency of activities with community organizations (≥11 h/week, 6–10 h/week, 3–5 h/week, 1–2 h/week, no community activities). Responses were categorized into four levels, as done in prior work (Kawachi et al., 1996; Loucks et al., 2006; Trudel-Fitzgerald, Poole, et al., 2019): socially isolated (individuals with low contacts—none or one of the following characteristics: married, had more than six close friends or relatives, attended weekly religious group activities, or attended weekly community activities [reference group]), moderately isolated (two characteristics), moderately integrated (three characteristics), and socially integrated (all four characteristics).

**References**

Berkman, L. F., & Syme, S. L. (1979). Social networks, host resistance, and mortality: A nine-year follow-up study of Alameda County residents. *American Journal of Epidemiology, 109*(2), 186-204. https://10.1093/oxfordjournals.aje.a112674

Chasan-Taber, S., Rimm, E. B., Stampfer, M. J., Spiegelman, D., Colditz, G. A., Giovannucci, E., . . . Willett, W. C. (1996). Reproducibility and validity of a self-administered physical activity questionnaire for male health professionals. *Epidemiology, 7*(1), 81-86. https://10.1097/00001648-199601000-00014

Chiuve, S. E., Fung, T. T., Rimm, E. B., Hu, F. B., McCullough, M. L., Wang, M., …Willett, W. C. (2012). Alternative dietary indices both strongly predict risk of chronic disease. *Journal of Nutrition, 142*(6), 1009-1018. https://10.3945/jn.111.157222

Chiuve, S. E., Rexrode, K. M., Spiegelman, D., Logroscino, G., Manson, J. E., & Rimm, E. B. (2008). Primary prevention of stroke by healthy lifestyle. *Circulation, 118*(9), 947-954. https://10.1161/CIRCULATIONAHA.108.781062

Giovannucci, E., Colditz, G., Stampfer, M. J., Rimm, E. B., Litin, L., Sampson, L., & Willett, W. C. (1991). The assessment of alcohol consumption by a simple self-administered questionnaire. *American Journal of Epidemiology, 133*(8), 810-817. https://10.1093/oxfordjournals.aje.a115960

Grimmett, C., Bridgewater, J., Steptoe, A., & Wardle, J. (2011). Lifestyle and quality of life in colorectal cancer survivors. *Quality of Life Research, 20*(8), 1237-1245. https://10.1007/s11136-011-9855-1

Holt-Lunstad, J., Robles, T. F., & Sbarra, D. A. (2017). Advancing social connection as a public health priority in the United States. *American Psychologist, 72*(6), 517-530. https://10.1037/amp0000103

Kawachi, I., Colditz, G. A., Ascherio, A., Rimm, E. B., Giovannucci, E., Stampfer, M. J., & Willett, W. C. (1996). A prospective study of social networks in relation to total mortality and cardiovascular disease in men in the USA. *Journal of Epidemiology and Community Health, 50*(3), 245-251. https://10.1136/jech.50.3.245

Kushi, L. H., Doyle, C., McCullough, M., Rock, C. L., Demark-Wahnefried, W., Bandera, E. V., …Gansler, T. (2012). American Cancer Society Guidelines on nutrition and physical activity for cancer prevention: Reducing the risk of cancer with healthy food choices and physical activity. *CA: A Cancer Journal for Clinicians, 62*(1), 30-67. https:// 10.3322/caac.20140

Loef, M., & Walach, H. (2012). The combined effects of healthy lifestyle behaviors on all cause mortality: A systematic review and meta-analysis. *Preventive Medicine, 55*(3), 163-170. https:// 10.1016/j.ypmed.2012.06.017

Loucks, E. B., Sullivan, L. M., D'Agostino, R. B., Sr., Larson, M. G., Berkman, L. F., & Benjamin, E. J. (2006). Social networks and inflammatory markers in the Framingham Heart Study. *Journal of Biosocial Science, 38*(6), 835-842. https://10.1017/S0021932005001203

McCullough, M. L., Feskanich, D., Stampfer, M. J., Giovannucci, E. L., Rimm, E. B., Hu, F. B., …Willett, W. C. (2002). Diet quality and major chronic disease risk in men and women: Moving toward improved dietary guidance. *American Journal of Clinical Nutrition, 76*(6), 1261-1271. https://10.1093/ajcn/76.6.1261

Mosca, L., Benjamin, E. J., Berra, K., Bezanson, J. L., Dolor, R. J., Lloyd-Jones, D. M.,…Wenger, N. K. (2011). Effectiveness-based guidelines for the prevention of cardiovascular disease in women 2011 update: A guideline from the American Heart Association. *Journal of the American College of Cardiology, 57*(12), 1404-1423. https://10.1016/j.jacc.2011.02.005

Rimm, E. B., Giovannucci, E. L., Stampfer, M. J., Colditz, G. A., Litin, L. B., & Willett, W. C. (1992). Reproducibility and validity of an expanded self-administered semiquantitative food frequency questionnaire among male health professionals. *American Journal of Epidemiology, 135*(10). https://10.1093/oxfordjournals.aje.a116211

Rimm, E. B., Stampfer, M. J., Colditz, G. A., Chute, C. G., Litin, L. B., & Willett, W. C. (1990). Validity of self-reported waist and hip circumferences in men and women. *Epidemiology, 1*(6), 466-473. https://10.1097/00001648-199011000-00009

Schlesinger, S., Walter, J., Hampe, J., von Schonfels, W., Hinz, S., Kuchler, T., . . . Nothlings, U. (2014). Lifestyle factors and health-related quality of life in colorectal cancer survivors. *Cancer Causes and Control, 25*(1), 99-110. Retrieved from https://10.1007/s10552-013-0313-y

Stampfer, M. J., Hu, F. B., Manson, J. E., Rimm, E. B., & Willett, W. C. (2000). Primary prevention of coronary heart disease in women through diet and lifestyle. *New England Journal of Medicine, 343*(1), 16-22. https://10.1056/NEJM200007063430103

Trudel-Fitzgerald, C., James, P., Kim, E. S., Zevon, E. S., Grodstein, F., & Kubzansky, L. D. (2019). Prospective associations of happiness and optimism with lifestyle over up to two decades. *Preventive Medicine, 126*, 105754. https://10.1016/j.ypmed.2019.105754

Trudel-Fitzgerald, C., Poole, E. M., Sood, A. K., Okereke, O. I., Kawachi, I., Kubzansky, L. D., & Tworoger, S. S. (2019). Social integration, marital status, and ovarian cancer risk: A 20-year prospective cohort study. *Psychosomatic Medicine, 81*(9), 833-840. https://10.1097/PSY.0000000000000747

Trudel-Fitzgerald, C., Tworoger, S. S., Poole, E. M., Williams, D. R., & Kubzansky, L. D. (2016). Prospective changes in healthy lifestyle among midlife women: When psychological symptoms get in the way. *American Journal of Preventive Medicine, 51*(3), 327-335. https://10.1016/j.amepre.2016.04.021
